# The Role of Day Care in the Provision of Integrated Services for Older People

**DOI:** 10.5334/ijic.9052

**Published:** 2025-07-17

**Authors:** Ailsa M. Cameron, Laura Bennett, Joanna Thorn, Demi Patsios

**Affiliations:** 1 Centre for Research in Health & Social Care, School for Policy Studies, University of Bristol, Bristol, UK; 2 Bristol Medical School (PHS), University of Bristol, Bristol, UK

**Keywords:** day care, voluntary sector, integration, older people

## Abstract

**Introduction:**

Day care services have a long history in the support of older people, both internationally and in England. However, despite their longevity there is no consensus about the aims of day care services and their contribution to integrated care is unrecognised. This paper defines day care as community building-based services that provide care and/or health related services and/or clubs and activities specifically for older people (65+) with care and support needs, which support wellbeing and health, and/or support people to remain living at home and/or enable informal carers to sustain care.

**Methods:**

The paper draws on qualitative data collected in a national study to reimagine day care services for older people in England. Eight sites participated, all from the voluntary sector.

**Results:**

Day care services act as both a site of, and facilitator to, integrated care and whilst settings experienced many of the challenges faced by voluntary sector organisations, including a reliance on limited and uncertain funding, they had developed a range of strategies to partially manage them.

**Conclusions:**

Collective forms of day care services have the potential to play a more significant role in the provision of local place based integrated care in England.

## Introduction

The move to greater integration has been a persistent theme of policy and practice in England, as well as internationally, over recent decades [1,2,3]. Whilst there is a lack of clarity about what integration entails, it is generally understood that integration refers to developments within the health sector or between health and social care sectors. Significantly, Goddard [4] notes that in recent years these developments have broadened out to involve other sectors including the voluntary sector (e.g. the NHS Long Term Plan 2019). However, difficulties of involving the voluntary sector have been acknowledged, including a lack of appreciation of services currently provided by the sector and the need for support to engage with commissioning processes [5]. Despite a lack of conceptual clarity there is widespread consensus that integration aims to improve service outcomes and their cost-effectiveness by enhancing collaborative practice across professional and organisational boundaries by “removing duplication, avoiding care gaps and improving patient/user and informal carer experience” [6: p. 2]. Previous work exploring the role of integrated children’s services [7] drew on discussions of embeddedness to consider how organisations that are structurally embedded in networks are able to achieve key social outcomes by drawing on their positions within these networks as well as their reputations for trustworthiness, characteristics often imbued by voluntary sector organisations. Debate around what integrated care means is often focused on the organisational and structural aspects, less attention is given to the person-centred aspects, namely “to better co-ordinate care around people’s needs” [8: p. 2]. This article addresses that gap.

In England, the Health and Care Act 2022, required health, social care and other local services to come together and establish Integrated Care Systems, which aim to support collaboration and shared decision making between services to better meet the needs of local populations, as well as enable a focus on prevention and health inequalities. At the same time, guidance was published setting out expectations that part of this new system was establishing “place-based partnerships”. These are described as the “foundations of integrated care systems” [9: p. 3], where organisations within a community or neighbourhood work together to best understand and meet the needs of that community or neighbourhood [9]. In this regard community-based services, such as collective forms of day care, could play a significant role.

Day care is generally understood as a building-based community service providing care and/or health related services and or activities for older people [10]. The international literature reports that day care can be provided by a range of different ‘social institutions’ from the health or social care sectors [11]. However, in England day care services are predominantly based within the voluntary sector [10]. Whilst there may be a shared understanding of what day care is, there is little agreement on its aims [12]. In England there is widespread acceptance of two broad types of provision [10]. The first of these is ‘specialist’ provision supporting people living with specific diseases, such as dementia, or with multiple morbidities and usually involves the provision of specialist and personal care. The second type of provision is ‘generalist’ day care, these services are aimed at people with lower levels of need and have a preventative and social focus. Both forms of day care services offer the potential to integrate services to ensure a more seamless experience for those attending, as well as their carers. However, as a form of social care provision, research suggests that collective forms of day care have declined over recent years [10]. The reasons for this are complex but there is widespread agreement that the congruent nature of day care is at odds with the aspirations of the personalisation agenda which emphasises the importance of individualised rather than collective forms of support [13]. In addition, day care services are thought to have been disproportionately impacted by austerity measures, leading to a reduction in funding [14]. Consequently, the decline in provision, coupled with a lack of appreciation of its role, results in policy and practice largely ignoring the potential of these services to address contemporary challenges, including the need to enhance integrated care, within the social care system [15].

The voluntary sector has always played a role in the provision of social care services in England [16]. However, its role has increased since the introduction of the 1990 NHS and Community Care Act. This legislation led to a reconfiguration of the care landscape, with a move away from statutory provision towards a mixed economy of care in which private and voluntary sector organisations are commissioned by local authorities to provide services. As a result of these changes, the voluntary sector, both national and local organisations, were estimated to be providing 17% of adult social care by in 2012 [17], although up-to-date figures are not available.

There is a widespread belief that the voluntary sector provides high quality services that are flexible and responsive to people’s needs and aspirations whilst at the same time being efficient and effective [18]. Voluntary sector organisations are also thought to be able to innovate quickly in response to changing circumstances, a characteristic that was widely lauded during the Covid-19 pandemic [19]. Recent research suggests that the actual market share of the voluntary sector in respect of adult residential care has “actually diminished over time relative to the for-profit growth” [20: p. 34], although it is unclear how this relates to community-based services. Indeed, research indicates that voluntary sector organisations remain vulnerable to funding cuts [21], and that community-based social care services are particularly at risk to a withdrawal or reduction in funding [14]. Importantly these cuts to adult social care services have a wider impact, not just on those relying on their support but also to the sector itself leading to a weakening of networks and partnerships and ultimately diminishing the organisational memory of how to work across organisational boundaries to support people living independently [22]. Whatever the impact of austerity measures it is widely accepted that the future of social care requires ‘a vibrant voluntary and community sector’ to contribute to supporting people to live independently in the community [23].

## Methodology

This paper reports findings from a mixed methods study reimagining collective forms of day care for older people in England. The study aimed to contribute to broader discussions about the current and potential role of local authority funded day care in meeting policy objectives across the social care and health sectors. Detail of the methodology has been published elsewhere [24]. Ethical review was provided by the National Social Care Research Ethics Committee, reference20/IEC08/0038. This paper presents data collected from 8 qualitative case-studies. The sites were originally to be sampled on the basis of geographic spread, organisational type including charities, statutory providers and private sector and, that they demonstrated some form of innovation in practice. Fieldwork took place between September 2021 and July 2022.

At each setting we aimed to interview participants with a range of perspectives: older people attending services (called members); carers; paid care workers and volunteers, as well as managers. In total we conducted 120 interviews: members (n = 37), carers (n = 10), care workers (n = 28), managers and finance leads (n = 15), volunteers (n = 10) and, stakeholders (n = 20). Interviews with members and carers explored experiences of day care including activities undertaken as well as perspectives on the support they received. Interviews with those working in, or managing, services focused on how services operated, including how they worked with other agencies, including health and social care partners as well as other community organisations. Interviews with local stakeholders including commissioners of adult social care services, health partners including specialist dementia services and other referral agencies, including social prescribers. These interviews focused on the place of day care provision in the local landscape of services and the potential contribution of day care provision both locally and nationally. Additional interviews were also conducted with staff and members of two centres run by and for minority ethnic communities and at a charity supporting LGBTQ+ people over the age of 50.

### Analysis

Interviews were audio recorded and transcribed in full. Analysis of the data was informed by the thematic analysis approach developed by Braun and Clarke [25]. A sample of 8 transcripts were read and independently coded by two members of the research team and a coding frame developed through discussion. Codes emerged inductively and deductively and were grouped in themes, these reflected the wider day care literature, integrated care and the challenges faced by the voluntary sector. The analysis focused on developing themes across the case study sites as well as explaining the differences between them. The process of analysis was managed using NVivo (12).

### The Involvement of People with Lived Experience

The research was supported by a reference group, comprising older people who attended a social club, the group met three times during the study. The group was facilitated by our practice partner and helped shape our proposed methods, the areas we were interested in exploring and our initial findings. In addition the advisory group included representation from people with lived experience.

## Findings

This paper reports key findings related to the place of collective forms of day care in the provision of integrated care for older people, focusing on their voluntary sector characteristics. However, we begin by setting out key characteristics of the participating sites and the day care they provide.

## Characteristics

### The organisations

The eight sites recruited (see Table 1) provided a range of models of day care. For example, our sites included an adapted farm for men living with Parkinson’s and activities offered at a community café. Despite trying to recruit day care sites from across different sectors, all of the organisations that agreed to take part in this study were based in the voluntary and community sector. One of the sites was run by a national charity, five were run by small scale local charities and two were community interest companies (a type of limited company that exists to benefit the community rather than shareholders). Five of the day care sites had existed for more than 10 years, while the remaining three had been set up more recently, to meet a specific need for day care provision in the wider community. For example, site 4 was set up to offer small scale, bespoke day care to people living with dementia in rural areas where access to services was limited. Four of the sites provided generalist day care, 3 specialist day care and the final site provided both, although these were based at different locations. Two of the specialist day care sites employed both qualified care workers and support workers, whilst generalist sites mainly employed support workers.

**Table 1:** Details of sites.


SITE	SPECIALISM	LOCALITY	ORGANISATION	MEMBERSHIP (APPROXIMATE ATTENDEES PER WEEK)	BRIEF DESCRIPTION

1.	Dementia	Urban	Local charity	250 members	Based in a Victorian villa, range of activities including music and art. Personal care available. Health colleagues ran specialist clinics with additional integrated services.

2.	Parkinson’s/dementia	Rural	Local charity	25 members	An adapted farm providing ‘agricultural type activities’ including feeding animals and tending vegetables. Minimal contact with statutory services but some cross referral. Open to men only

3.	General activities & support	Urban	Local charity	30 members	Based in a purpose built centre. Provides a range of traditional activities including bingo, quizzes and exercise sessions. Health partners funded various preventative services.

4.	Dementia	Rural & urban	Community Interest Company (CIC)	330 members	Based in a ‘hosts’ home, for groups of 4–5 adults, activities include preparing lunch, quizzes and crafts. Collaborative working and cross referral took place.

5.	General activities & support	Urban	Local charity	4000 members	Based in multiple repurposed centres. Provides an extensive programme of activities in partnership with external organisations including singing and exercise groups. Funded to provide community element of some NHS care pathways. Inclusive policies and practice observed.

6.	General activities & support	Urban	CIC	20 members	Based in a repurposed building in a public park. Provides a community café for all and a lunch club and group activities.

7.	General activities for faith community with specific provision for people with visual difficulties	Urban	Local charity	140 members	Based in a purpose built centre. Provides a range of traditional activities including art, conversation groups, entertainment and culturally specific activities. Faith-based centre


### The day care offer

Whilst the ‘offer’ of each setting was different, there were broad similarities. For example most, but not all, sites offered a range of different activities that members attending could choose to take part in, these were generally structured before or after a shared social meal. The range of these activities are discussed in later sections. At three sites the ‘offer’ was rather different. For example, site 6 offered a limited range of stand-alone groups arranged around a lunch club, these included an intergenerational social group, a carers group and crafting sessions. At site 2, the adapted farm, the day was arranged around a series of farming tasks, for example preparing and distributing food for animals, tidying the farmyard and tending to vegetables. Members usually choose the task they most enjoyed, but if they choose not to participate then alternative tasks were available. Pre-lockdown, site 2 had organised the day around a shared meal but had ended this practice due to logistical difficulties when reopening as well as ongoing concerns about cost.

### The experiences of members and carers

Across sites, all of the members we spoke to were positive about their experience of day care. In the main they valued the opportunities to socialise with other people. For some people attendance at day care was an opportunity to meet old friends and/or make new friends, contributing to improved sense of wellbeing and mitigating loneliness and isolation. For example, a member at site 3 commented “so, it’s the companionship and having somebody to talk to, and sharing their experiences and having a laugh” (S03OP07). Many of the members appreciated the opportunity to take part in ‘purposeful’ activities for example learning new skills, such as life drawing or using existing skills such as woodwork. These opportunities appeared to build self-esteem and confidence and contributed to improved wellbeing. One member explained the value in attending cookery classes as “It’s not just to learn to prepare something new. It’s to be together because again, the social aspect is very important in my view. It’s not just what you practice there and then. It’s the banter. We joke. We talk about politics, about football. Then we eat a meal” (S05OP04).

Carers were particularly appreciative of day care services. They viewed day care as an essential support that enabled them to maintain their caring function. A carer at site 2, whose husband attended the farm twice a week, explained “it just enables me to go out and do shopping and occasionally see a friend or whatever. […] At the moment, that’s all I have and that’s okay” (S02C03). Carers appeared to have developed a high level of trust in the staff working in the day care services who were often a consistent point of contact in an unpredictable world. A carer at site 4 described, “It’s sort of useful because when he’s not here I’m not worried because I know he’s safe and he’s enjoying himself. […] I know he’s safe, I know he can’t come to any harm. Therefore, I’m not worried” (S04C02). Carers also spoke about the additional support, both emotional and practical, they received. A carer at site 3 described how the manager “[…] was always on hand for when I needed information about getting [mum] a blue badge, getting her different benefits, etc” (S03C01). Time and again the relationships built up with staff appeared to bolster the experience of carers and suggested that a more relational approach to the work undertaken within day care settings, beyond the ‘task’ of caring, was important.

## Integrated care

### Working with health partners

Despite a perception that community-based social care services can struggle to work closely with health partners [26,27], most of the centres in this study described working closely with a range of organisations. Not surprisingly specialist day care sites had well developed links illustrating their formal integration with the wider health sector. For example, the manager at site 1 described working closely with a range of health professionals and services including the continence service, safeguarding services and doctors at the local dementia clinics. The close nature of these working relationships was emphasised by a stakeholder who described how, “I, as a care co-ordinator and one of our consultants actually hold a clinic often at [site 1]. We try to do it every couple of months, just to run through service users that we’ve got at the day centre just to kind of like reinforce support is there and for us to review and feedback” (SO1SH02). The importance of ‘informal’ integrated working practices were also a feature at specialist sites. A host at site 4, the small-scale day care service for people living with dementia, described how, because of the pressures on district nurses she had an important role in ensuring services were aware of any changes amongst those who attended. She explained “so we are the eyes and ears of what’s going on, particularly, for the ones that live alone [….] (S04CW04).”

There were examples of both formal and informal integrated working practices at generalist day care sites. For example, site 5 was commissioned by NHS partners to run exercise classes as part of the community element of a clinical falls pathway. A stakeholder described “I think it meets the evidence-based practice of having someone engage in a strength and balance programme for an extended period of time […]. Pragmatically, it’s great because we’re one of the few areas in [city] and possibly across the UK that does have that onward pathway of doing an NHS programme with the service and then going into something like [site 5]” (S05SH010). In addition, one of the benefits of this approach was that having completed the community element of the programme at the setting, people were more likely to engage in other physical activities based there, demonstrating a wider preventative role. This scheme was thought to be having a positive impact on the sector, a care worker commented “I definitely think the evidence is there, that if you have got organisations like [site 5] you are reducing the use of the health service and social care. […] That is a massive big saving because you are keeping people out of hospital” (S05CW05).

At all of the sites the relational skills of the staff were key to the effectiveness of both the formal and informal approaches to integrated working [28]. In particular, the long-term, trusted relationships developed by staff with members, their carers and families enhanced their preventative role. As one care worker described, “It’s also, to a certain extent, a monitoring process, a wellbeing process. Somebody spending a good amount of time with them, three or four hours. Because district nurses and that, they’re so pressurized, they’re in and out like a yo-yo, and so are day to day carers. […] and also, because very few of these agencies manage to get consistency with the same person. So, actually, if you’ve got a complete stranger doing it, they don’t know if something’s changed” (S04CW04). This point was echoed by a stakeholder at site 3 who noted the same restrictions on health and care professionals in contrast to day care staff “[…] that’s one of the massive, massive benefits of these types of services, that because they know the individuals well, and usually, the families as well, they can pick up when there are problems, before it becomes a crisis” (S03SH02).

There were also examples of collaborative working with health partners at a strategic level. For example, several stakeholders noted that the CEO of site 3 was heavily involved in local strategic partnerships which had raised the profile of the organisation and led to it receiving funding from local GPs to run a range of preventative sessions including diabetes awareness and exercise sessions. The CEO described the importance of this collaborative approach, “I attend care management meetings, which is collectively with GPs, the intermediate care team and our social worker team. […] When it comes down to it, we’ve all got the same agenda, and it’s about working together to provide the best options” (S03M01). Other sites were actively working on developing links with health partners. The manager at site 6 stated “we’ve got some really good relationships with GP practices. Some still don’t get it but a lot of them are starting to come around” (S06M01). However, wider appreciation of the role of collective day care amongst health partners helped to build the contribution these services could play within integrated services.

### Local connections

Like many voluntary sector organisations, all of the sites appeared to be well embedded within their local communities. This included having an understanding of the local community and their needs, as one stakeholder describes “… the innovations, as I say, hopefully coming from just listening to their communities, hearing what their communities want, reflecting the communities that are around them and being there for that diverse community” (S03SH01).

Most day care organisations had strong links with multiple community organisations including local schools, faith groups and residential care facilities and several had links with cultural organisations including libraries and galleries. These relationships served several purposes. For example, the manager at site 3 described the range of organisations they had links with and the benefits of these which included being seen as an important community resource, she described “I know it sounds crazy, but the local undertakers, the local florists, they will signpost people to us. […] So it’s about building that understanding in the community. But, yes, from police, hospitals, social workers, social prescribers, GPs, pharmacists, to the local Asda” (S03M01). These relationships worked in both directions, ensuring that staff were able to refer members and their carers to a range of community organisations or services when needed. A care worker at site 5 described “frequently there are other issues, whether its housing, or it could be memory loss, or it could be they’re struggling emotionally, and they did have some support but they could do with some more, we need to make another referral” (S05CW02). Having knowledge of local services and organisations was crucial in supporting members and carers and demonstrates the importance of these embedded relationships to the work of centres. They also suggest an implicit appreciation of the importance of collaboration that may be distinct to the voluntary sector.

Having extensive contacts within the community also helped centres develop the range of activities available to members. Several of the sites had links with local theatres and galleries which enabled them to put on a wide range of cultural activities. Site 5 worked with an array of sports clubs, including a community gym, and used these to extend their range of regular activities, as one member described “And some of the sports activities that I participate in […] they’re actually done outside, because [site 5] have partnerships with other organisations. So, they’re in partnership with some local bowls clubs” (S05OP01).

Having strong local connections meant that managers at several sites were seen as key figures within the local voluntary sector, helping to shape and influence local decisions. A stakeholder at site 6 described how these local connections gave the centre credibility within the sector and enabled the manager to advocate on behalf of the community. They commented “I think, as an organization, they take stock of what they’re actually seeing and what they’re hearing on a day-to-day basis. […]. That will help [the manager] identify if there’s a gap or a local need […]. He does a lot of partnership meetings with other voluntary sector groups and statutory groups. He comes to a lot of council meetings where we’re understanding if there are any gaps […], if anything has just suddenly come to the forefront and has been highlighted. Then we just look to pull something together, really, as a partnership” (S06SH01). The ability of day care to lead collective responses was demonstrated during the national lockdown introduced in response to Covid-19. During this period several of the sites acted as a community hub, facilitating community and statutory organisations to work together [24].

### Collaboration versus competition

In addition to these benefits managers and stakeholders at several sites commented that collaborative working between voluntary sector organisations was an effective strategy particularly when funding was scarce. A stakeholder at site 3 suggested that collaborations within the voluntary sector were helpful because “[…] it could be that you’d have where organisations are competing against each other on a particular bid that they would benefit from doing more of a joint-bid approach to guarantee securing that funding and then reaching their communities” (S03SH01). The finance manager at this site endorsed this view suggesting “[…] and that’s where I feel significant partnership work comes about, it’s about sharing resources and skills where there isn’t an additional cost” (S03F01). However, whilst collaboration was seen as beneficial, the manager at site 6 recognised that sometimes organisations were unable to work together or chose not to, he said “Some of that is around just the day to day, the demand, the capacity […] some of it is around competition, history” (S06M01).

## Challenges Facing Day Care Services

Not surprisingly all of the sites reported facing significant challenges, some of which could undermine integrated working. There were many different aspects to these challenges and whilst some reflected the specific context of the social care sector, particularly the financial difficulties, others appeared to relate to their status as voluntary sector organisations.

### Funding

The most pressing challenge facing day care related to funding. All of the sites participating in the study relied on external funding to support their work. The ways in which they received funding varied. For example, site 1 owned the building the centre was based in and received grant funding from the clinical commissioning group (CCG) to support various activities. The finance manager explained, “[w]e get a central grant of about £98,000 a year, which is what they call a core grant, which is given towards the salary costs”. They went on to explain that originally this had covered the total cost of all salaries, but that it had been reduced “and it represents about 18% of our total income” (S01F01). Site 1 was also contracted to provide care for members referred via the local authority who had been assessed as eligible for publicly funded care, of whom there were currently 17. The remainder of their members were self-funders. Finally, the finance manager reported relying on additional pots of funding including from charities as well as using government funding to support staff training. As a specialist provider, site 1’s position appeared to be more secure than some of the generalist day care centres taking part, but there was still a sense of uncertainty and concern about the precarity of funding.

Site 2 was also a specialist centre, supporting men living with Parkinson’s disease and/or dementia however, their funding situation was very different, they received a small amount of funding from statutory sources and relied more heavily on charitable foundations and bequests. The reasons for this were complex but reflected previous experience of working with local authority commissioners who were perceived to be overly bureaucratic and ultimately undermining of the spirit of the adapted farm, for example placing a greater emphasis on health and safety which limited the ethos of the site. The manager explained her reluctance to work more closely with the local authority, saying “I think we could face more regulations, if it goes like the young people [services]. So, you could face more regulations where you’re not able to do this unless you’re registered with the CQC [Care Quality Commission] and everything has to be more formal” (S02M01). Although some members were self-funders and a small number used their direct payment to fund their attendance the organisation’s reluctance to engage more extensively with statutory services meant they relied on raising charitable funds which carried its own risks. The manager explained, “It could be that we don’t exist anymore, because we just can’t keep going, I just can’t do it, we can’t fight for the funding and that could happen, I guess. That’s the risks” (S02M01). The constraining nature of contracting with local authorities was acknowledged by a stakeholder who remarked “But I do sometimes think that those links with the public sector, whilst still being really important, they’ve also limited some organisations” (S02SH03).

All of the generalist sites reported having a patchwork of funding. The manager at site 5, the large scale day care provider that operated across two local authorities, described how their income was split into 5 categories. These were: contracts with local authority partners (adult social care and public health) which represented the largest source of income; adult community learning funding; health partners; trusts and foundations and finally, members fees and donations. Although site 5 had always received funding from health partners this had declined in recent years. The manager went on to say “I think it’s going to be an interesting conversation for [Site 5] over the next year, but trying to highlight the fact that everyone thinks we do a good job, everyone sees we’ve got a place in the health and care system, but health have decided they’re not going to give us any money; that doesn’t seem right” (S05M01).

The precarity of funding from the statutory sector was common across all sites. Indeed, many noted closures of day care organisations in their local areas. The manager at site 8 described that “the two centres that did cater for all those [additional] needs, […] they closed down. […] I think the impact has been that those people are becoming more isolated and more alone, and the pressures on the carers has increased” (S08SH04). Several participants suggested that commissioners were not fully cognisant of the fragility of the voluntary sector. For example, a stakeholder at site 1 argued that whilst voluntary sector organisations were flexible and adaptive to changes in the wider context, this flexibility came with some risks, they said “It’s trying to get the council and the CCG to understand that these organisations do an awful lot with very little funding and they hold huge risks as well. There’s a hell of a lot of work that people do that doesn’t get recognised. It can be quite scary that there’s sometimes a real divorce between what they think is actually happening and what is actually happening […]” (S01SH01). Despite this difficult picture the CEO at site 4 told us that some commissioners understood the risks taken by the voluntary sector and provided financial support, she explained how the organisations recent expansion into a new locality would not have happened “unless we’d had pump priming from [the] County Council” (S04M01).

### Bureaucratic burden

Receiving funding from statutory bodies led to increased levels of bureaucracy which if not kept in check could impact core business. These processes included completing risk assessments, having to submit regular activity reports and responding to retendering exercises. The manager at site 5 described the increasing burdens of these processes, he said “[o]ur commissioners, quarterly is what they’re looking for. And we will tend to report on […] number of users, number of unique users, number of activity hours and number of activities. Increasingly, we are being asked for more sophisticated data than that, which […] we don’t have the systems in place to be able to give” (S05M01). These processes weighed heavily on all of the day care sites and as we have discussed, led one site to reduce their reliance on statutory sector funding. This is an important point and suggests the need for commissioners to consider the proportionality of the bureaucracy associated the commissioning process, particularly on voluntary sector providers.

## Discussion

The findings presented in this paper demonstrate that voluntary sector day care centres are uniquely placed to play a role in achieving government ambitions for a greater focus on preventative care as part of local place-based partnerships [9]. Several were formally operating as sites of integrated care practice, either providing specific programmes of activities that were funded by health partners, including as part of an existing NHS care pathway or, providing venues for health partners to hold regular clinics in order to review the circumstances of individual members. In addition, staff at all of the sites acted as facilitators of integrated care, referring members and their carers to other services when circumstances dictated or contacting health or social care partners when the needs of members changed. However, a key question underpinning this article is whether the status of participating sites, as voluntary sector organisations, supports or hinders their role in terms of the provision of integrated services.

Reflecting previous research [7], the degree to which organisations were embedded within their local community appeared to influence their positions within the local health and social care system. All of the centres had strong links with a range of statutory and voluntary sector organisations, as well as with other community organisations and these relationships were critical to staff ability to refer people to appropriate support when needed. In addition, their understanding of the needs of their communities, borne as a result of long-term relationships with the people who attended and their families, meant that they enjoyed a degree of credibility both within the voluntary sector but also amongst statutory partners. This credibility was bolstered by their reputation for collaborative working which was enhanced during Covid-19. In recognition of this reputation, managers from several of the organisations were regularly invited to represent the voluntary sector in different forums with statutory partners. Attendance at these meetings appeared to raise the profile of day care, further adding to the sense in which they were recognised as influential partners in the integrated agenda and potentially opening-up further opportunities.

An important feature of their ability to facilitate integrated services for members and their carers appeared to rest on the relational skills of individual staff working within the sites. The data revealed that staff, and their managers, were highly valued by members and their carers who turned to them as trusted sources of support. This finding echoes previous work by Henderson et al., [26] who argue that trusting relationships are a cornerstone of integrated practice. Arguably it was the informality of settings that appeared to lend itself to staff spending more time with members, getting to know them and valuing these relationships. The nature of these relationships, often built up over years, appeared to stand in stark contrast to those experienced with staff working in statutory services where the pressures resulting from austerity were thought to be most acute. This is an important finding because it suggests that staff working in day care settings are well placed to attend to the person-centred aspects of integration, ensuring that services are co-ordinated around people’s needs [8].

All of the sites demonstrated creativity in terms of the partnerships they had developed with other community organisations, a characteristic widely assumed as a feature of the reputation of the voluntary sector [29]. These partnerships helped to enhance the range of activities day care centres offered and therefore improved the experience of those attending. However, the push to innovate also reflected the financial constraints facing the social care sector as well as the need to respond to external events like the Covid-19 pandemic [19]. Consequently, it is important to acknowledge Chew and Lyon’s [29] warning, that being innovative can bring its own challenges which can require organisations to take further risks. In this sense day care organisations faced a double jeopardy related to the conditions that required them to innovate and the additional risks associated with innovation. These risks could potentially be amplified if commissioners either did not fully appreciate the risks or chose not to understand them.

Analysis of the data revealed that whilst all of the sites appreciated the need for cooperation, a key element of integrated practice and a hallmark of voluntary sector values [18], there was some evidence of tensions emerging between voluntary organisations. Indeed, several managers implied they operated a contingent approach to working closely with other voluntary organisations with whom they may be competing for funding from the statutory sector. This finding is not surprising particularly given the difficult financial circumstances facing the social care sector. It demonstrates the fragility of relationships within the quasi market and suggests the need for a more nuanced approach to commissioning services, one that supports mutuality rather than undermining it through the contracting process. In addition, it was clear that relationships with statutory partners were fraught, often as a direct result of the additional bureaucratic tasks associated with receiving funding from the statutory sector, including having to respond to local authority requirements to retender a service. There were also concerns about the potential erosion of the social values underpinning the ethos of the organisation [29] and to the innovative model of day care that several of the sites were operating. This impact is particularly harmful if government wishes to encourage the greater involvement of the voluntary sector in the provision of integrated care and potentially undermines the new approach ‘to collective public service provision’ [19: P. 464] that was evident in response to Covid-19. Whilst the findings point to the need for commissioners to foster a new relationship with the voluntary sector per se, they also suggest that commissioners require a more nuanced appreciation of the distinct nature of collective forms of day care within the wider social care landscape, and particularly their current contribution, as well as their potential contribution, to integrated care.

## Limitations

It was originally intended that sites would be sampled on the basis of organisational type and that they demonstrated some form of innovation in practice. However, the impact of lockdown and the closure of day centres meant that selecting sites was more difficult than anticipated because many had removed detailed information about activities from web-sites or had closed. Consequently all of the sites were based within the voluntary sector and in addition, the range of innovatory practice may be limited.

## Conclusions

As we have demonstrated the voluntary sector plays a significant role in the provision of day care services for older people in England and, that in this role, they act as a site of integrated care and a facilitator of integrated care practice. However, this role is poorly understood and rarely acknowledged, consequently their potential contribution to integrated care systems is not harnessed. The data presented suggests that the embedded nature of the day care organisations participating in this study, the long-term nature of their relationships with members, as well as their carers and, the ability of organisations to innovate were important factors to this work. In addition, the data reveals that the organisations faced many of the challenges known to impede the role of voluntary sector when working with local authorities, including precarious funding and burdensome bureaucratic processes. Given the difficulties facing the social care sector, it is important as Mayrhofer et al., [22] have argued, that the role of the voluntary sector is recognised and supported to ensure its sustainability. This may necessitate a more nuanced approach to commissioning, one that supports the fragile nature of the voluntary sector. This is vital for maintaining the valuable sense of community that members and their carers view as an important part of their lives.
